# Effect of hypocaloric versus normocaloric parenteral nutrition on whole body protein kinetics in critically ill neurosurgical patients

**DOI:** 10.1186/cc12189

**Published:** 2013-03-19

**Authors:** O Rooyackers, A Berg, J Wernerman

**Affiliations:** 1 Karolinska Institutet and University Hospital, Huddinge, Sweden

## Introduction

The optimal feeding of critically ill patients treated in the ICU is controversial. Present guidelines for protein feeding are based on weak evidence obtained with suboptimal methods. Whole body protein kinetics is an attractive technique to assess optimal protein intake by measuring the effect of protein feeding strategies on protein synthesis rates, protein degradation rates and protein balance. Here protein kinetics were measured in critically ill neurosurgical patients during hypocaloric and normocaloric parenteral nutrition.

## Methods

Neurosurgical patients on mechanical ventilation (*n = *16) were studied. Energy expenditure was measured with indirect calorimetry. After that, the patients were randomized to receiving 24 hours of 50% of measured energy expenditure followed by 24 hours of 100% or 100% before 50%. Whole body protein kinetics were measured during the last half hour of the feeding periods using stable isotope-labeled phenylalanine as a tracer. During a continuous infusion of labeled phenylalanine and tyrosine, plasma samples were obtained and later analyzed for the content of the labeled amino acids using mass spectrometry. Protein kinetics were calculated using standard steady-state kinetics. In addition, amino acid concentrations were analyzed by HPLC. Student's *t *test was used for statistical analyses.

## Results

The patients received 0.5 ± 0.1 and 1.1 ± 0.2 g amino acids/ kg/day (*P <*0.001) on the days with 50 and 100% of measured energy expenditure respectively. Energy expenditures were 23.4 ± 2.7 and 24.5 ± 2.3 kcal/kg/day (*P *= 0.05) on the 50 and 100% days respectively. Plasma amino acids concentrations were 2.8 ± 0.5 and 2.9 ± 0.4 mM (*P *= 0.085) on the 2 days respectively. Whole body protein synthesis was 12% lower when 50% of energy expenditure was given, 11.7 ± 3.0 versus 13.3 ± 2.2 mg/kg/hour (*P *= 0.025), whilst protein degradation was unaltered 13.6 ± 3.5 versus 14.0 ± 2.6 mg/kg/hour (*P *= 0.56). Also protein oxidation was unaltered 3.0 ± 2.1 versus 2.9 ± 1.4 mg/kg/hour (*P *= 0.85). This resulted in a 60% higher whole body protein balance with the normocaloric nutrition, -1.9 ± 2.1 versus -0.7 ± 1.3 mg/kg/ hour (*P *= 0.014).

## Conclusion

The protein kinetics measurements and the protocol used were useful to assess the efficacy of nutritional support in critically ill patients. In the critically ill neurosurgical patients treated in the ICU, hypocaloric feeding was associated with a more negative protein balance, while the amino acid oxidation was not different.

